# Surgery of the Sternum

**Published:** 1899-01

**Authors:** 


### SURGERY OF THE STERNUM.

Longuet (*Progress Medical*, July 23, 1897) gives a summary of the surgery of the sternum as follows: (1) Sternectomy for affections of the sternum. This may be indicated (1) exceptionally in congenital malformations causing compression of the lung; (2) in traumatic osteitis and abscess, and for bullets lodged in the posterior table of bone; (3) for osteitis which may be pyogenic, tuberculous, syphilitic, or due to actinomycosis; (4) for tumors—enchondroma and sarcoma, most of the latter being beyond treatment owing to their rapid growth and extension. The author divides the operations into atypical, consisting of partial operations for osteitis and sequestrotomy, and typical resections for tumor. Partial superior sternectomy may be done by Bardenheuer's method, which preserves a bridge of bone between the clavicles. A vertical incision is made down the center of the manubrium, and a second between the sterno-clavicular joints. Each clavicle is separated from the first rib, and 1 cm. of the latter resected on each side; the manubrium is then divided transversely 1 cm. below its upper border and removed. The normal attachment of the clavicles is thus preserved. Dudon's operation differs in removing whole manubrium after dividing the first two costal cartilages on each side and opening the sterno-clavicular joints. The aim of Bardenheuer's operation is to prevent falling in of the clavicles and constriction of the subjacent organs; but Longuet denies that there is any proof that this occurs. Movement of the arms after the operation of complete removal of the manubrium is said to be good, the clavicles not being indispensable. The complications are: (1) Wounds of the jugular and innominate views, causing hemorrhage and air entry; (2) wounds of the pleura, causing pneumothorax; (3) wounds of the pericardium. (4) Sternectomy for operations on the mediastinum. This may be required (1) for foreign bodies, (2) mediastinal suppuration, (3) aneurism, (4) new growths. Bardenheuer and Tabrulay have performed partial resection of the manubrium as a preliminary to ligation of the innominate artery for aneurism. For benign new growths, such as lipoma, fibroma and cysts, temporary resection is required, but for malignant tumors permanent resection is necessary. In operations for tumors Longuet does not think Bardenheuer's operation gives enough room for manipulation, and prefers complete removal of the manubrium.—*British Medical Journal.*

